# *Bacillus velezensis* QSE-21 cell-free supernatant primes resistance and outperforms live cells in controlling *Botrytis cinerea* on tomato

**DOI:** 10.3389/fmicb.2025.1639396

**Published:** 2025-08-07

**Authors:** Saisai Gao, Hongjia Han, Fan Yang, Xinyang Liu, Wenxing Liang, Mengjie Liu

**Affiliations:** ^1^Key Laboratory of Integrated Crop Pest Management of Shandong Province, College of Plant Health and Medicine, Qingdao Agricultural University, Qingdao, China; ^2^College of Life Sciences, Shandong Normal University, Jinan, China

**Keywords:** *Bacillus velezensis*, cell-free supernatant, tomato gray mold, transcriptomics, immune priming

## Abstract

**Introduction:**

Biological control agents (BCAs) offer an environmentally friendly alternative to chemical pesticides for plant disease management. However, the efficacy of live microbial BCAs is often compromised by ecological constraints. Cell-free supernatants (CFSs), derived from BCA fermentation, contain most active biocontrol compounds responsible for disease suppression and can be directly applied without introducing living organisms into the environment. Our prior work demonstrated that CFS from *Bacillus velezensis* QSE-21 (CFS-Q) directly inhibits the growth and development of *Botrytis cinerea*. This study investigates CFS-Q-induced systemic resistance in tomato plants and fruits against *B. cinerea*.

**Methods:**

Tomato seedlings were foliar-sprayed with CFS-Q or controls. Systemic resistance was assessed by challenging distal leaves with *B. cinerea*. Comparative transcriptomics analyzed gene expression (RNA sequencing) in treated vs. untreated plants, with/without pathogen inoculation. Tomato fruits were sprayed with CFS-Q, live QSE-21 cells (Cell-Q), or LB medium (control group), followed by *B. cinerea* inoculation.

**Results:**

Application of CFS-Q triggered immune responses in tomato seedlings, conferring enhanced local and systemic resistance against *B. cinerea* without direct pathogen contact. Comparative transcriptomics revealed that CFS-Q treatment activated multiple immune signaling pathways in tomato, regardless of *B. cinerea* inoculation. This immune priming effect translated into significantly faster and stronger defensive reactions against *B. cinerea* attack. Crucially, compared to spraying live QSE-21 cells, spraying CFS-Q exhibited superior efficacy in controlling *B. cinerea* on tomato fruits.

**Discussion:**

CFS-Q operates via a dual mechanism: direct antagonism (established previously) and induced systemic resistance (ISR), evidenced by immune pathway activation. The priming effect ensures rapid defense mobil.

## 1 Introduction

The fungal pathogen *Botrytis cinerea*, responsible for plant gray mold disease, is capable of infecting over 1,000 plant species, including more than 200 agriculturally important crop cultivars (Elad et al., 2016; Richards et al., 2021). Due to its broad host range, potent destructive capability, and capacity to cause substantial crop and economic losses, *B. cinerea* ranks among the 10 most important fungal plant pathogens (Dean et al., 2012; Soltis et al., 2019). The prevention and control of gray mold have long been performed mainly by using chemical pesticides (Fillinger and Walker, 2016). While chemosynthetic pesticides have played a critical role in controlling gray mold and other plant diseases, the disadvantages associated with their use have become increasingly apparent (Wang et al., 2018). The extensive application of chemical pesticides has led to several issues, such as environmental pollution, the development of drug resistance, and the presence of pesticide residues in agricultural products (Rahman et al., 2024). Consequently, there is an urgent need to develop new strategies for plant disease management that reduce reliance on chemical pesticides (Jaiswal et al., 2022; Rahman et al., 2024).

In recent years, the quest for alternatives to chemical fungicides has highlighted the potential of biological control agents (BCAs) to be an increasingly attractive option (Zhang et al., 2024). Various types of BCAs have been successfully used for the management of plant diseases (Rahman et al., 2024). BCAs with antagonistic microorganisms as active ingredients have the advantages of high safety, environmental friendliness, and sustainability (Jiang et al., 2015). Unlike chemical pesticides, microbial antagonists do not induce resistance after long-term use, primarily due to their multiple antibacterial mechanisms. Microbial antagonists can exert biocontrol effects through direct actions such as site competition, nutrient competition, and the production of antibiotics, as well as indirect effects, like promoting plant growth and inducting plant immunity (Nicot et al., 2016; Shan et al., 2024). Multiple types of antagonistic microorganisms can activate the immune system through a phenomenon known as induced system resistance (ISR) in plants (Figueredo et al., 2017; Kaneko et al., 2023). ISR leads to a primed resistance state in which plants exhibit more rapid and robust activation of various defense responses upon attack by harmful organisms (Conrath et al., 2015; Mauch-Mani et al., 2017; Ngoc Huu et al., 2022). Antagonistic microorganisms trigger plant defenses via various secreted signaling compounds, such as phytohormones, peptides, organic acids, and siderophores (Kumari et al., 2024). These signaling compounds enhance plant defenses via plant hormone signal transduction, mitogen-activated protein kinase (MAPK) signaling, intracellular calcium signaling, and activation of secondary metabolite production (e.g., phenolic compounds and phytoalexins via the phenylpropanoid pathway) (Kumari et al., 2024; Naeem et al., 2020).

Biological control offers several advantages and appears to be the best option for the development of low-cost, eco-friendly and sustainable management approaches for protecting plants and crops (Syed Ab Rahman et al., 2018). However, despite decades of extensive research, biological control methods remain less effective than chemical control methods in regulating plant health (Rahman et al., 2024; Singh et al., 2020). Although many BCAs demonstrate high efficacy under laboratory conditions, both *in vitro* or *in vivo*, their efficiency is strongly affected during field applications. Under complex natural environmental conditions, various factors, such as humidity, temperature, pH, and other abiotic and biotic stresses, impact the effectiveness of BCAs (Akum et al., 2021; Yang et al., 2019). Additionally, since biological control involves introducing live nonnative organisms, which may invade plants or have negatively affect the environment, there are potential risks of severe ecological impacts (Jennings et al., 2017; Singh et al., 2020). Therefore, it is an imperative to identify safer and more effective biological control measures to manage harmful organisms under natural conditions (Rahman et al., 2024; Vieira et al., 2022).

Compared to using live microbial pesticides, employing microbial metabolites to control plant pests does not require introducing nonnative living organisms, thereby posing a lower risk of uncontrollable biological effects. Moreover, microbial metabolites are more resilient during storage and easier to mix with other chemical or biological pesticides. Nevertheless, extracting and producing a single metabolite from microbial fermentation broth involves complex steps and is costly, hindering the practical implementation of microbial metabolites (Rahman et al., 2024). After microbial fermentation, numerous active substances are present in the cell-free supernatant (CFS) of the fermentation broth, which can effectively control pathogens (Xiao et al., 2021). For instance, the CFS of *Bacillus subtilis* ZD01’s fermentation broth exhibits nearly equivalent ability to control potato early blight compared to bacterial cells (Zhang et al., 2022). The fermentation filtrate of *Streptomyces* sp. JCK-6131 effectively suppresses the development of various pathogenic fungi and bacteria and activates plant immunity at low concentrations (Le et al., 2021). Similar functions have also been observed in the fermentation filtrate of other *Streptomyces* sp. strains (Nasr-Eldin et al., 2019; Patil et al., 2011; Vergnes et al., 2020). Additionally, stable indoor fermentation conditions are more conductive to producing active biocontrol substances compared to applying live BCAs in natural environments. Therefore, exploring the effects of the bacterial CFS and utilizing it as an effective agent represents a crucial direction for the development of biological control.

In our previous research, we reported that the CFS of *Bacillus velezensis* QSE-21, an endophyte isolated from a tomato plant, significantly inhibited the growth and development of *B. cinerea* (Xu et al., 2021). In this study, we further investigate the function and mechanism of QSE-21` CFS (CFS-Q) in inducing plant resistance. Initially, we detect a series of signaling events in tomato plants following treatment with CFS-Q. We also examine the resistance of tomato plants and fruit to *B. cinerea* induced by CFS-Q. Furthermore, a transcriptomic analysis is conducted to explore the gene transcription responses of tomato plants with or without *B. cinerea* infection after CFS-Q treatment. Additional analysis aims to understand the priming function of CFS-Q in activating multiple immune-related signaling pathways in tomato. Our findings enhance the understanding of how the endophytic bacterium QSE-21 induces plant immunity and offer a promising method for effectively managing gray mold disease in tomato plants and postharvest fruits using the fermentation broth of a BCA.

## 2 Materials and methods

### 2.1 Strains and preparing the CFS of QSE-21

*Bacillus velezensis* strain QSE-21 was previously isolated from tomato (*Solanum lycopersicum*) plant stem tissue and was deposited at the China Center for Type Culture Collection (CCTCC, Wuhan University, China) with an accession number of CCTCC M 2020295 (Xu et al., 2021). To prepare the cell-free supernatant (CFS) of QSE-21, the strain was streaked from −80°C freezer stocks onto LB agar plate, and grown overnight at 28°C. Single clone was picked into LB medium, cultured overnight at 28°C with shaking at 200 rpm, then diluted 1/100 into fresh LB medium and further incubated for another 3d on a shaker (180 rpm) at 25°C. The culture was centrifuged at 10,000 g for 15 min. The precipitate after centrifugation was washed three times with PBS, resuspended, and used as a live microbial agent (Cell-Q). The supernatant was filtered with a 0.22 μm filter membrane to remove the remaining bacteria and the filtrate was used as the CFS of QSE-21` fermentation broth (CFS-Q).

### 2.2 Tomato plant and fruit treatment by CFS-Q

The cherry tomato plants were grown in indoor climate chamber at 25°C with a 16/8 h day/night cycle, and 4 to 5-week-old plants were used in this study. For cell death induction, the true leaves were injected with 50 μL CFS-Q and observed within 12 h. For the detection of ROS, callose and Ca^2+^ ions, RNA-seq or *B. cinerea* infection, the CFS-Q was sprayed evenly on the detached or living leaf or postharvest fruits surface using a small sprayer. After naturally air-drying, the treated leaves or plants or fruits were returned to their original growth condition or used for subsequent experiments.

### 2.3 Histochemical detection of H_2_O_2_, O^2–^, callose deposition and intracellular Ca^2+^ ions

The accumulation of H_2_O_2_ and O^2–^ in tomato leaves were determined with nitroblue tetrazolium (NBT) and 3,3′-diaminobenzidine (DAB) histochemical staining methods, respectively, according to previous methods (Liu et al., 2015). The callose was stained with aniline blue solution and observed with an epifluorescence microscope under the UV channel, according to previous method (Ton and Mauch-Mani, 2004). The intracellular Ca^2+^ ions were visualized by the fluorescent Ca^2+^ indicator Fluo-3 AM (ThermoFisher, F14218), according to previously described method (Hwi Seong et al., 2022). After stained by the indicator, the fluorescence signal was observed with an epifluorescence microscope under the green (G) channel.

### 2.4 *Botrytis cinerea* infection and disease assessment

For detached leaves and postharvest fruits, 10 μL droplets of *B. cinerea* at 1 × 10^6^ conidia ml^−1^ was inoculated to the surface to evaluate disease symptoms. The inoculated leaves or fruits were placed in high-relative-humidity conditions (c.95%) at 25°C with a 16/8 h day/night cycle. Disease lesion area in 10–15 leaves per treatment was calculated by measuring the lesion diameter using a cross method. For living leaves, each of 5 μL droplets of *B. cinerea* at 1 × 10^6^ conidia ml^−1^ was inoculated on 15–20 fully grown leaves per plants. The inoculated plants were carefully placed in high-relative-humidity conditions (c.95%) at 25°C with a 16/8 h day/night cycle. Disease symptoms were evaluated by determining the mean lesion diameter in each leaf. Symptoms from the *B. cinerea* infected leaves were classified into 5 classes as previously described (Ngoc Huu et al., 2022) with slight modifications. The symptoms were classified as: Class I, lesion diameter < 2 mm; Class II, lesion diameter 2–5 mm; Class III, lesion diameter 5–10 mm; Class IV, lesion diameter 10–15 mm; Class V, lesion diameter > 15 mm to the entire leaf are affected. Three replicates were conducted, each using 30–40 leaves from 2 plants.

### 2.5 RNA extraction, RT-qPCR and transcriptome sequencing

Tomato seeding leaves or flesh tissue of fruits from different treatment groups were collected and powdered in liquid nitrogen and then used for total RNA extraction. Total RNA was extracted using TRIzol reagent (Invitrogen, Carlsbad, CA, United States) according to the manufacturer’s instructions. For reverse transcription quantitative PCR (RT–qPCR), 1 μg of total RNA of each sample was used for cDNA synthesis and then used for qPCR, according to the previously description (Hongjia et al., 2022). The transcriptional level of genes was calculated according to the 2^−ΔΔCq^ method (Kenneth and Thomas, 2001) using tomato actin gene (SlACT, Solyc04g011500) as endogenous reference. Primers used in RT–qPCR were designed by Beacon Designer software and were listed in Supplementary Table S1. For transcription sequencing, 2 μg of total RNA per sample was used for Illumina library construction by ApexBio Technology LLC. (Shanghai) following the recommendations provided by the manufacturer. After quality-assessing using an Agilent 4200 Bioanalyzer (Agilent, USA), the library was sequenced using the Illumina Xplux sequencing platform (Paired end150) to generate raw reads. The raw data have been deposited in the National Center for Biotechnology Information (NCBI) Sequence Read Archive (SRA),^1^ and the accession number is PRJNA1215988.

### 2.6 RNA-seq raw data analysis, genes annotation and cluster analysis

Raw data analysis and genes annotation were conducted by ApexBio Technology LLC. (Shanghai) according to standard procedures (Kukurba and Montgomery, 2015). Briefly, raw paired-end fastq reads were filtered by Fastp (Chen et al., 2018) discard the adapters and low quality bases. The clean reads obtained were then aligned to the *S. lycopersicum* reference genome SL3.0 (EnsemblPlant, GCA_000188115.3) using HISAT2 (Kim et al., 2015), followed by reference genome-guided transcription assembly and gene expression quantification using StringTie (Pertea et al., 2015). Differentially expressed genes (DEGs) were identified by DEseq2 (Love et al., 2014) with a cut-off value of log2|fold-change| > 1 and p-adjust <0.05. Function enrichment analysis for DEGs based on Gene Ontology (GO) and Kyoto Encyclopedia of Genes and Genomes (KEGG) pathway categories was performed using the clusterProfiler 4.0 (Wu et al., 2021). Terms with *p*-value < 0.05 were considered significant. Cluster analysis of DEGs was performed and visualized as heatmap using the online platform^2^ SRplot (Tang et al., 2023).

### 2.7 Activity measurement of defense-related enzymes

Activity of the defense-related enzymes, including phenylalanine ammonialyase (PAL), polyphenoloxidase (PPO), β-1,3-glucanase (GLU), and chitinase (CHI), were assayed using the kits (PAL, BC0210; PPO, BC0190; GLU, BC0365; CHI, BC0825) from Solarbio science & technology (Beijing) Co., Ltd. The assays were conducted according to the manufacturer’s protocols. All the enzyme activities were calculated on the basis of fresh weight (FW), which were expressed as U/g FW. All measurements were performed in triplicates with each sample collected from three biological replicates.

### 2.8 Statistical analysis

Statistical analyses were performed using GraphPad Prism software. The data were obtained from replicates per experimental condition and expressed as mean ± standard deviation. Significant differences between experimental groups were evaluated by one-way ANOVA with Tukeys HSD test. Statistical significance was set at *p* < 0.05. All experiments were repeated at least 3 times with similar results.

## 3 Results

### 3.1 CFS-Q induces plant immune responses

**Figure 1:**
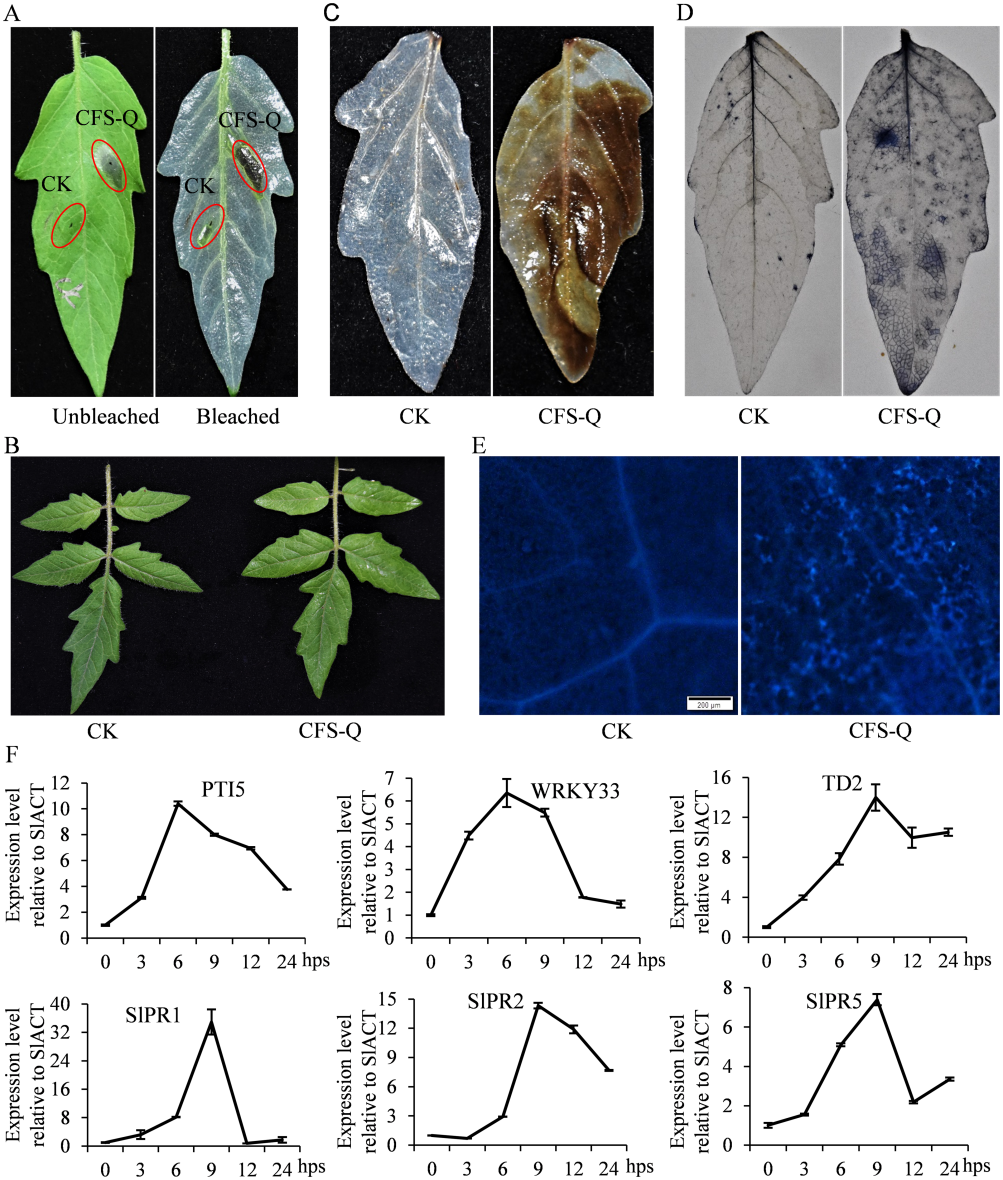
CFS-Q treatment induced tomato immune responses. **(A)** Injection of CFS-Q induced cell death in tomato leaves. **(B)** Spraying with CFS-Q induced no visible changes in tomato leaves. Detection of H_2_O_2_ **(C)**, O_2_^–^ **(D)**, and callose deposition **(E)** in tomato leaves with or without CFS-Q treatment. **(F)** The relative expression levels of the indicated genes were determined via RT–qPCR, with SlACT used as an endogenous reference. The expression level of each gene at 0 hps was set as “1” and used as a sample control.

A previous study revealed that the CFS of *B. velezensis* QSE-21 fermentation broth (CFS-Q) could activate the JA pathway in tomato fruit (Xu et al., 2021), indicating that CFS-Q may also function in inducing plant immunity. After being injected into tomato leaves, CFS-Q induced strong and rapid cell death within 6 h post infection (hpi; Figure 1A). After being evenly sprayed on tomato leaves, CFS-Q caused no visible changes in the treated leaves (Figure 1B). Six hours post spraying (hps), the tomato leaves were stained with DAB, NBT or methyl blue. In contrast with the leaves treated with LB medium, the leaves treated with CFS-Q were dark brown and navy in color (Figures 1C,D), indicating that spraying with CFS-Q caused the accumulation of ROS (H_2_O_2_ and O_2_^–^) in the leaves. Under an epifluorescence microscope with a UV filter, fluorescent spots were visible on the leaves treated with CFS-Q (Figure 1E), indicating that spraying with CFS-Q caused callose deposition. Transcript levels of plant immunity and PR marker genes rapidly increased after CFS-Q treatment (Figure 1F). Most of the tested genes maintained high expression levels after CFS-Q treatment within 24 hps, and the expression levels peaked at 6 or 9 h after CFS-Q treatment (Figure 1F).

### 3.2 CFS-Q improves tomato resistance to *Botrytis cinerea*

**Figure 2:**
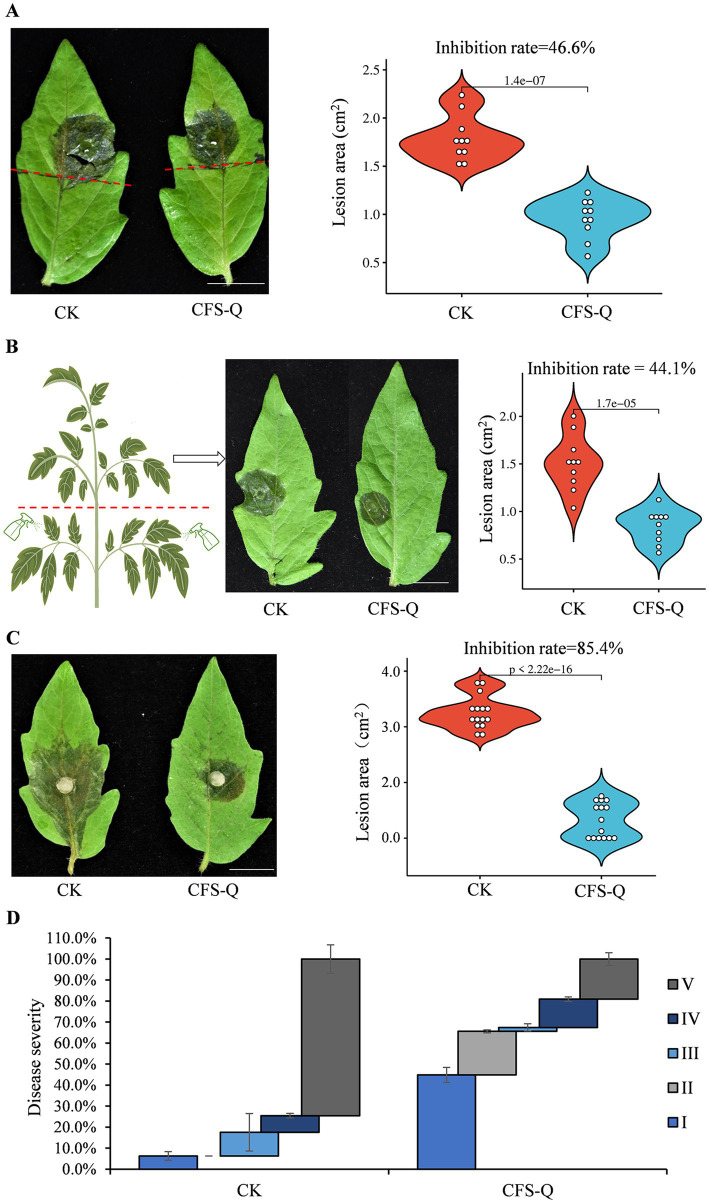
CFS-Q treatment improved tomato resistance to *B. cinerea*. **(A)** Disease phenotype and lesion size of tomato leaves pretreated on the lower half. **(B)** Disease phenotype and lesion size of tomato leaves from the upper branches of seedlings pretreated on the lower branches. **(C)** Disease phenotype and lesion size of detached tomato leaves from seedlings subjected to uniform pretreatment of the entire plant. **(D)** Disease severity caused by *B. cinerea* in live tomato leaves. Bar = 1 cm. Tomato leaves or seedlings pretreated with LB culture were used as the CK group.

A previous study revealed that CFS-Q had a significant inhibitory effect on hyphal growth and spore germination in *B. cinerea* (Xu et al., 2021). To eliminate the direct inhibitory effect of CFS-Q on *B. cinerea* growth, the lower halves of tomato leaves or the lower branches of tomato plants were treated with CFS-Q, and *B. cinerea* conidia were inoculated onto the upper halves of these leaves or leaves in the upper branches (not sprayed with CFS-Q) to avoid direct contact with CFS-Q. After incubation under moist conditions for 72 h, the lesions from *B. cinerea* infection on CK leaves or plants (treated with LB medium instead of CFS-Q) were larger than those on CFS-Q-treated leaves or plants (Figures 2A,B). The lesion areas of ten leaves from the CK and CFS-Q treatment groups were measured. The average lesion areas of leaves and plants from the CK group were approximately 1.8 cm^2^ and 1.5 cm^2^, respectively, whereas those of the leaves and plants of the CFS-Q treatment groups were approximately 0.9 cm^2^ and 0.8 cm^2^, respectively. The inhibition rates of the CK and CFS-Q treatment groups were approximately 46.6 and 44.1%, respectively (Figures 2A,B).

To investigate the biocontrol potential of CFS-Q against *B. cinerea* on plants, 6-week-old tomato seedlings were sprayed with CFS-Q or LB medium (CK), after which the *B. cinerea* spores were inoculated onto detached or live leaves. After incubation under moist conditions for 72 h, all detached leaves in the CK group were diseased, with an average lesion area of 4.5 cm^2^, whereas only approximately 60% of the CFS-Q-treated leaves were diseased, with the largest lesion area being only 1.5 cm^2^ (Figure 2C). The inhibition rate of CFS-Q against *B. cinerea* was approximately 85.4% (Figure 2C). After incubation under moist conditions for 72 h, the distribution of disease symptoms in live leaves from tomato plants infected with *B. cinerea* was analyzed. More than 64% of the tomato leaves in the CFS-Q treatment group presented class I and class II disease symptoms, whereas more than 80% of the tomato leaves in the CK group presented class IV and class V disease symptoms (Figure 2D). These results indicate that plants treated with CFS-Q presented significantly improved resistance to *B. cinerea*.

### 3.3 Gene expression levels in tomato plants were altered by CFS-Q treatment and *Botrytis cinerea* infection

**Figure 3:**
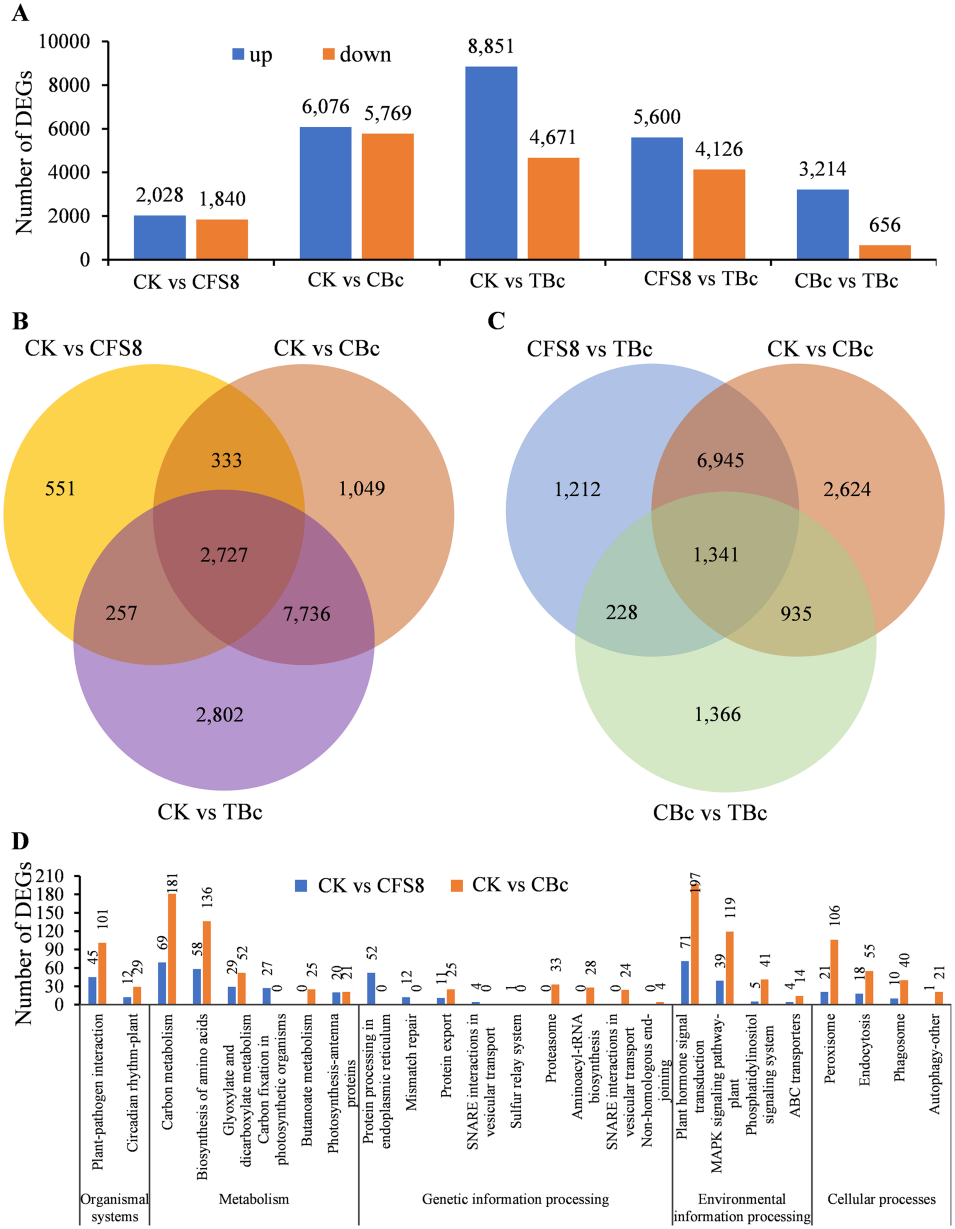
Overview of differentially expressed genes (DEGs) identified in tomato leaves from different treatment groups. **(A)** Number of DEGs identified in the indicated groups. **(B,C)** Venn diagram showing commonly and specifically expressed genes in tomato leaves subjected to different treatments. **(D)** Number of DEGs in the CK vs. CFS8 and CK vs. CBc comparisons annotated to the top 20 significantly enriched KEGG pathways and functional classification analysis. CK, leaf sample without treatment. CFS8, leaf sample collected at 8 h posttreatment with CFS-Q. CBc, leaf sample collected at 24 h postinoculation with *B. cinerea*. TBc, Leaf sample collected at 24 h post inoculation with *B. cinerea* from leaves pretreated with CFS-Q for 8 h.

A transcriptomic analysis was conducted to investigate the expression levels of tomato genes under CFS-Q treatment and *B. cinerea* infection. Tomato leaves from four different treatment groups were collected: leaves not sprayed with CFS-Q (CK), leaves 8 h after spraying with CFS-Q (CFS8), CK group leaves inoculated with *B. cinerea* for 24 h (CBc), and CFS8 group leaves inoculated with *B. cinerea* for 24 h (TBc). Differentially expressed genes (DEGs) were identified using thresholds of adjusted *p* value (Padj) < 0.05 and at least a 2-fold change (|log2FC| > = 1) in the normalized fragments per kilobase of exon model per million mapped fragments (FPKM) values. Compared with CK, both CFS-Q treatment and *B. cinerea* infection led to a significant number of upregulated and downregulated genes in tomato leaves (Figure 3A). A total of 3,868 DEGs were found in the CK vs. CFS8 comparison, with a majority shared with the CK vs. CBc comparison (3,060 DEGs) and the CK vs. TBc comparison (2,984 DEGs) (Figure 3B). Venn diagram analysis revealed 2,727 DEGs shared among all three comparisons (Figure 3B). While most DEGs were common between the CK vs. CBc and CFS8 vs. TBc comparisons, there were 1,440 unique DEGs in the CFS8 vs. TBc comparison and 3,559 unique DEGs in the CK vs. CBc comparison (Figure 3C). A total of 3,870 DEGs were identified in the CBc vs. TBc comparison, with the majority (3,214/3,870) being upregulated (Figure 3A). Venn diagram analysis revealed 1,569 DEGs and 2,276 DEGs in the CBc vs. TBc comparison were shared with the CFS8 vs. TBc comparison and CK vs. CBc comparison, respectively, and that 1,341 DEGs were shared among all three comparisons (Figure 3C). These findings suggest that CFS-Q pretreatment partially altered the impact of *B. cinerea* infection on tomato gene expression.

GO and KEGG pathway enrichment analyses were performed to identify the functional classifications of the DEGs found in the CK vs. CFS8 and CK vs. CBc comparisons. The top 20 significantly enriched terms were visualized in a scatter plot. Notably, most of the top 20 significantly enriched terms were consistent between the CK vs. CFS8 and the CK vs. CBc comparisons (Supplementary Figure S1). Most of the GO terms were related to the formation of cellular structure, such as thylakoid, cell membrane, and some GO terms were related to carbohydrate metabolism and photosynthesis (Supplementary Figure S1). In the KEGG pathway and functional classification analysis, the top 20 significantly enriched KEGG pathways in the CK vs. CFS8 comparison and CK vs. CBc comparison were assigned to five main categories (Figure 3D). As shown in the histogram, most of the KEGG pathways were consistent in the CK vs. CFS8 comparison and the CK vs. CBc comparison (Figure 3D). Notably, some KEGG pathways related to plant immunity and ISR, such as “plant–pathogen interaction,” “plant hormone signal transduction,” “MAPK signaling pathway–plant,” and “peroxisome,” were significantly enriched in both the CK vs. CFS8 and CK vs. CBc comparisons (Figure 3D).

### 3.4 CFS-Q treatment activated multiple immune-related signaling pathways in tomato

**Figure 4:**
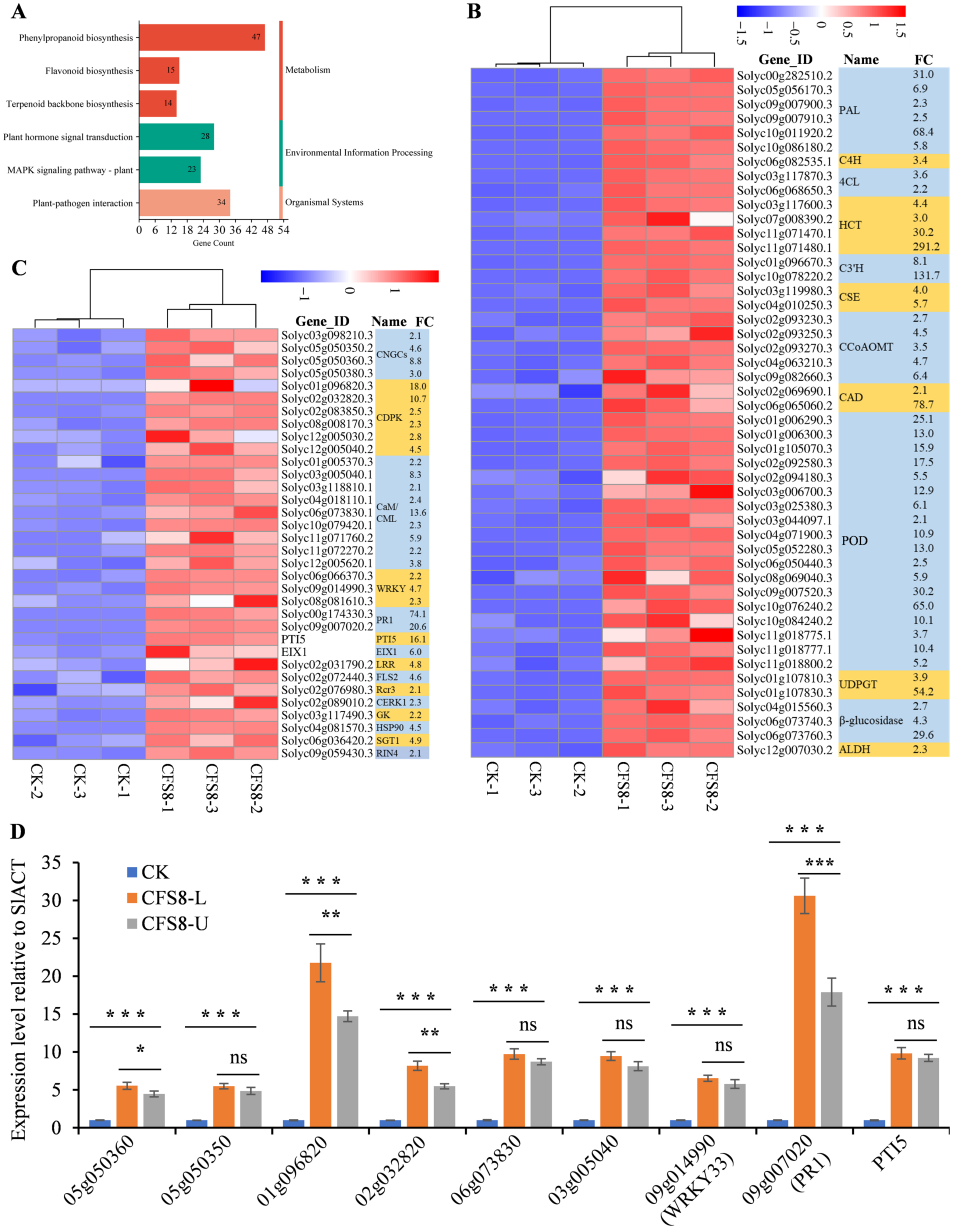
CFS-Q treatment activates immune-related signaling pathways in tomato leaves. **(A)** CFS-Q upregulated genes enriched in six KEGG pathways related to plant resistance. The numbers on the bar chart represent the number of upregulated genes annotated to this pathway. **(B,C)** Heatmap showing the RNA-seq expression levels of the upregulated genes annotated to the phenylpropanoid biosynthesis pathway and plant–pathogen interaction pathway in tomato leaves with or without CFS-Q treatment. The normalized FPKM value of each replicate is shown in the heatmap. FC, fold change in CFS8/CK based on the average of the normalized FPKM values for three replicates. **(D)** The relative expression level of the indicated genes detected by RT-qPCR using SlACT as endogenous reference. The expression level of each gene in CK group was set as “1” and used as sample control. CK, leaf sample without treatment. CFS8, leaf sample collected at 8 h post treatment by CFS-Q. CFS8-L, leaf sample directly exposed to CFS-Q and collected at 8 h. CFS8-U, leaf sample indirectly exposed to CFS-Q and collected at 8 h. *p < 0.05; **p < 0.01; ***p < 0.001.

In the CK vs. CFS8 comparison, 531 upregulated genes were annotated to KEGG pathways. Six of KEGG pathways were related to plant resistance to pathogen infection, including the phenylpropanoid biosynthesis pathway, the plant–pathogen interaction pathway, the plant hormone signal transduction pathway, the MAPK signaling pathway-plant, the flavonoid biosynthesis pathway, and the terpenoid backbone biosynthesis pathway (Figure 4A). A total of 48 upregulated genes were annotated to phenylpropanoid biosynthesis pathways, mainly the lignin biosynthesis pathway (Figure 4B; Supplementary Figure S2). The enzymes catalyzing the first three reactions of phenylpropanoid biosynthesis (PAL, phenylalanine ammonia lyase; C4H, cinnamic acid 4-hydroxylase; and 4CL, 4-coumarate-CoA ligase) were significantly upregulated to varying degrees (Figure 4B). Several key enzymes involved in lignin biosynthesis, including hydroxycinnamoy-CoA shikimate/quinate hydroxycinnamoyl transferase (HCT), *ρ-*coumaroyl shikimate 3′-hydroxylase (C3′H), caffeoyl-CoA 3-O-methyltransferase (CCoAOMT), cinnamyl alcohol dehydrogenase (CAD), UDP-glucosyltransferases (UDPGTs), and peroxidases (POD), were significantly upregulated to varying degrees (Figure 4B). A total of 15 upregulated genes were annotated to the flavonoid biosynthesis pathway, but most were associated with the phenylpropanoid biosynthesis pathway (Figure 4B; Supplementary Figure S3). Furthermore, 4 chalcone synthases (CHSs), the first enzymes involved in flavonoid biosynthesis, were not significantly upregulated (Supplementary Figure S3). A total of 34 upregulated genes were annotated to the plant–pathogen interaction pathway and were associated mainly with calcium ion (Ca^2+^) signaling pathways, including cyclic nucleotide gated channel (CNGC), calcium-dependent protein kinase (CDPK), calmodulin and calmodulin-like protein (CaM/CML), and WRKY transcription factor (Figure 4C) signaling. A total of 28, 23, and 14 upregulated genes were annotated to the plant hormone signal transduction, MAPK signaling pathway-plant and terpenoid backbone biosynthesis pathways, respectively (Supplementary Figure S4).

To investigate the induced systemic resistance of CFS-Q in tomato, the expression levels of 6 representative DEGs in leaves either directly exposed to CFS-Q or indirectly exposed (the upper leaves of the plants with CFS-Q sprayed on the lower leaves) using RT-qPCR. Compared to the CK group (leaves sprayed with LB medium), all 9 genes in the directly treated leaves (CFS8-L) and the systemic untreated leaves (CFS8-U) showed significantly upregulated, and the folding changes of 4 genes in the CFS8-U sample were slightly lower than those in the CFS8-L sample (Figure 4D).

### 3.5 CFS-Q improved the activity of enzymes related to plant disease resistance

**Figure 5:**
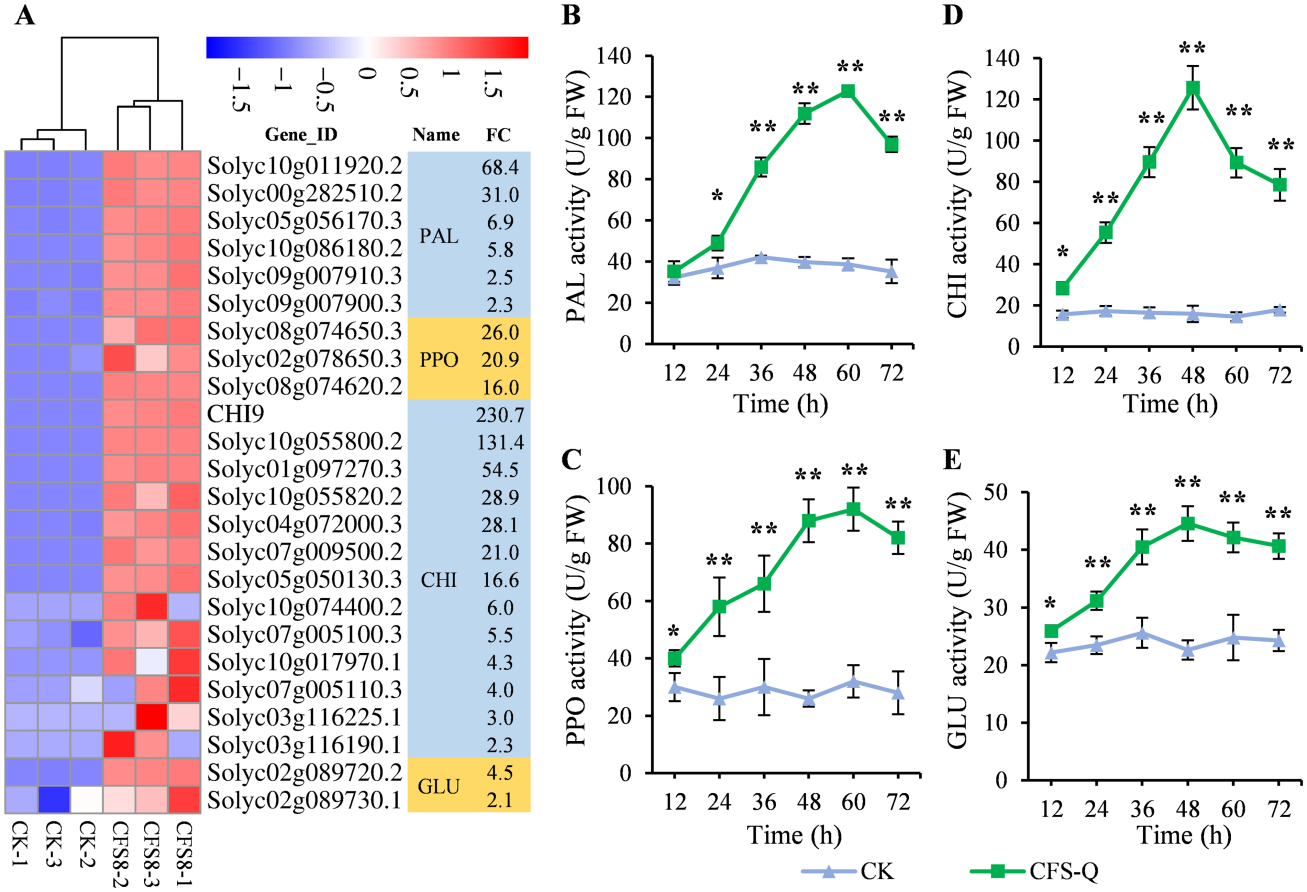
CFS-Q induced defense-related enzyme activities in tomato leaves. **(A)** Heatmap showing the RNA-seq expression levels of defense-related enzyme-encoding genes in tomato leaves with or without CFS-Q treatment. The normalized FPKM value of each replicate is shown in the heatmap. FC, fold change in CFS8/CK based on the average of the normalized FPKM value for three replicates. CK, leaf sample without treatment. CFS8, leaf sample collected at 8 h posttreatment with CFS-Q. **(B–D)** Changes in defense-related enzyme activities over time in tomato leaves with or without CFS-Q treatment. The enzyme activity units are expressed on a fresh weight basis as U g−1. *p < 0.01; **p < 0.001.

Defense-related enzymes, such as phenylalanine ammonia lyase (PAL), polyphenol oxidase (PPO), chitinase (CHI), and β-1,3-glucanase (GLU), play crucial roles in defense against pathogen infection in plants (Prabhukarthikeyan et al., 2018; Yan et al., 2021). The genes encoding these defense-related enzymes were screened from the RNA-seq data. A total of 24 genes, including 6 genes encoding PAL, 3 genes encoding PPO, 13 genes encoding CHI and 2 genes encoding GLU, were upregulated in the CK vs. CFS8 comparison (Figure 5A). The activities of these enzymes were measured, and the results are shown in Figure 5. Without treatment, the activities of the 4 enzymes did not significantly fluctuate at any of the time points tested (Figures 5B–E). In contrast, the activities of the four enzymes increased sharply in the CFS-Q-treated tomato leaves. The activities of PAL and PPO peaked at 60 h, and the activities of CHI and GLU peaked at 48 h. After the peak, the activities of these 4 enzymes in the CFS-Q-treated tomato leaves decreased but remained high compared with those in the untreated leaves (Figures 5B–E).

### 3.6 CFS-Q primed the intracellular Ca^2+^ concentration and strengthened the Ca^2+^ signaling pathway following *Botrytis cinerea* infection

**Figure 6:**
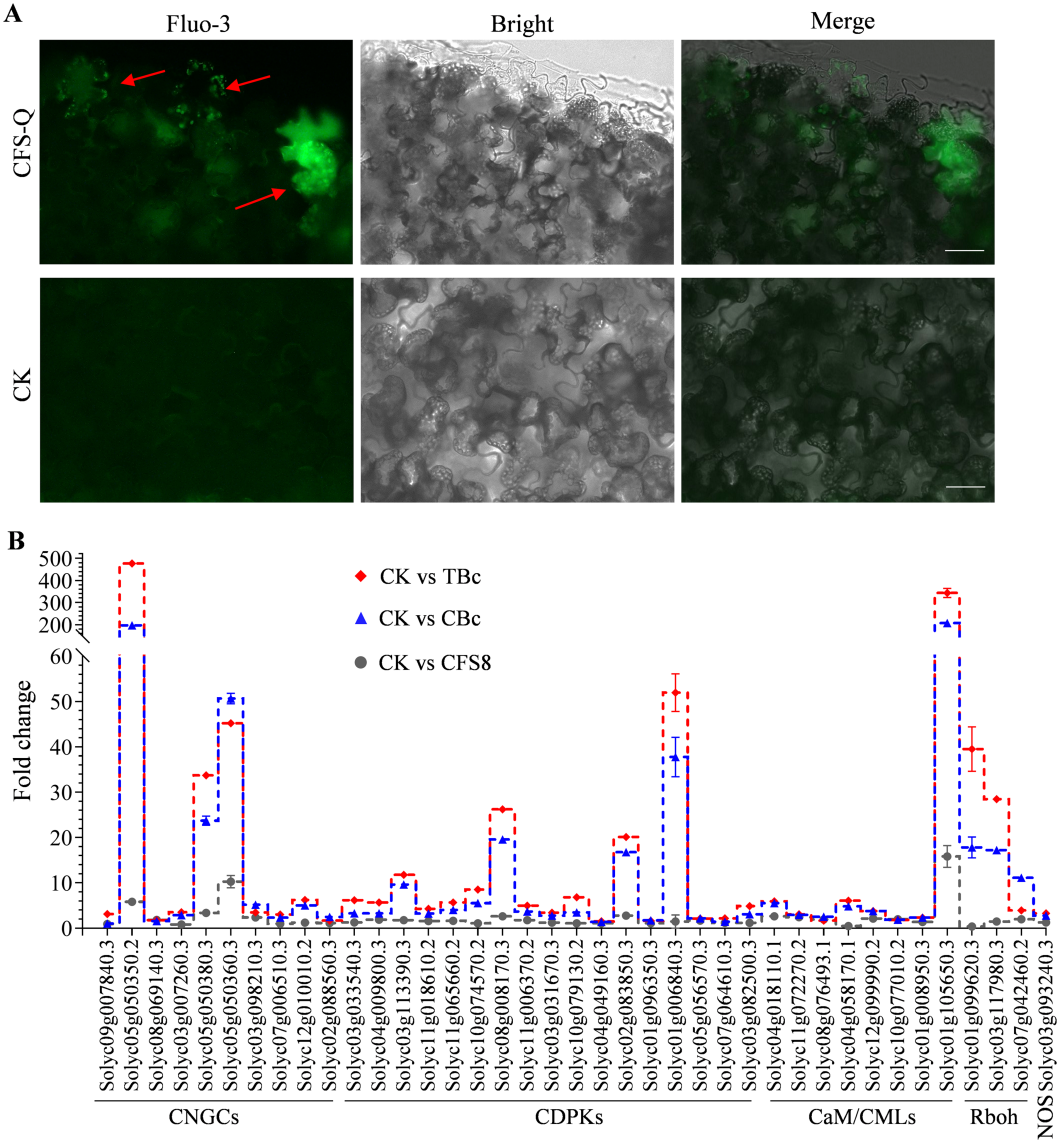
CFS-Q primes intracellular Ca^2+^ signaling. **(A)** Free Ca^2+^ in tomato leaves detected by Fluo-3 AM staining with or without CFS-Q treatment. Bar = 20 μm. **(B)** Relative expression levels of genes annotated to Ca^2+^ signaling under the indicated conditions. CK, untreated leaf sample. CFS8, leaf sample collected at 8 h posttreatment with CFS-Q. CBc, leaf sample collected at 24 h postinoculation with *B. cinerea*. TBc, leaf sample collected at 24 h post inoculation with *B. cinerea* from leaves pretreated with CFS-Q for 8 h. The fold change in the expression of the indicated genes was determined from RNA-seq data.

After CFS-Q treatment, the expression of several CNGC genes encoding a group of nonspecific Ca^2+^-permeable cation channels was upregulated (Figure 4C). Accordingly, the changes in [Ca^2+^]_cyt_ were determined using the fluorescent Ca^2+^ indicator fluo-3-acetoxymethyl ester (Fluo-3 AM). Compared with those in the untreated tomato leaf sections, more cells presented a dot fluorescence signal, and the fluorescence signal was more visible in the sections of the CFS-Q-treated leaves (Figure 6A). A total of 39 upregulated genes related to Ca^2+^ signaling (Supplementary Figure S5) were identified in the RNA-seq data (Figure 6B). Notably, most of the 39 genes (30/39) in the CK vs. TBc comparison presented higher upregulated expression levels than did those in the CK vs. CBc comparison (Figure 6B). These results indicate that CFS-Q pretreatment could prime and strengthen Ca^2+^ signaling in tomato to allow the plants to cope with *B. cinerea* infection.

### 3.7 The control efficacy of CFS-Q against *Botrytis cinerea* in tomato fruits was superior to that of living microorganism

**Figure 7:**
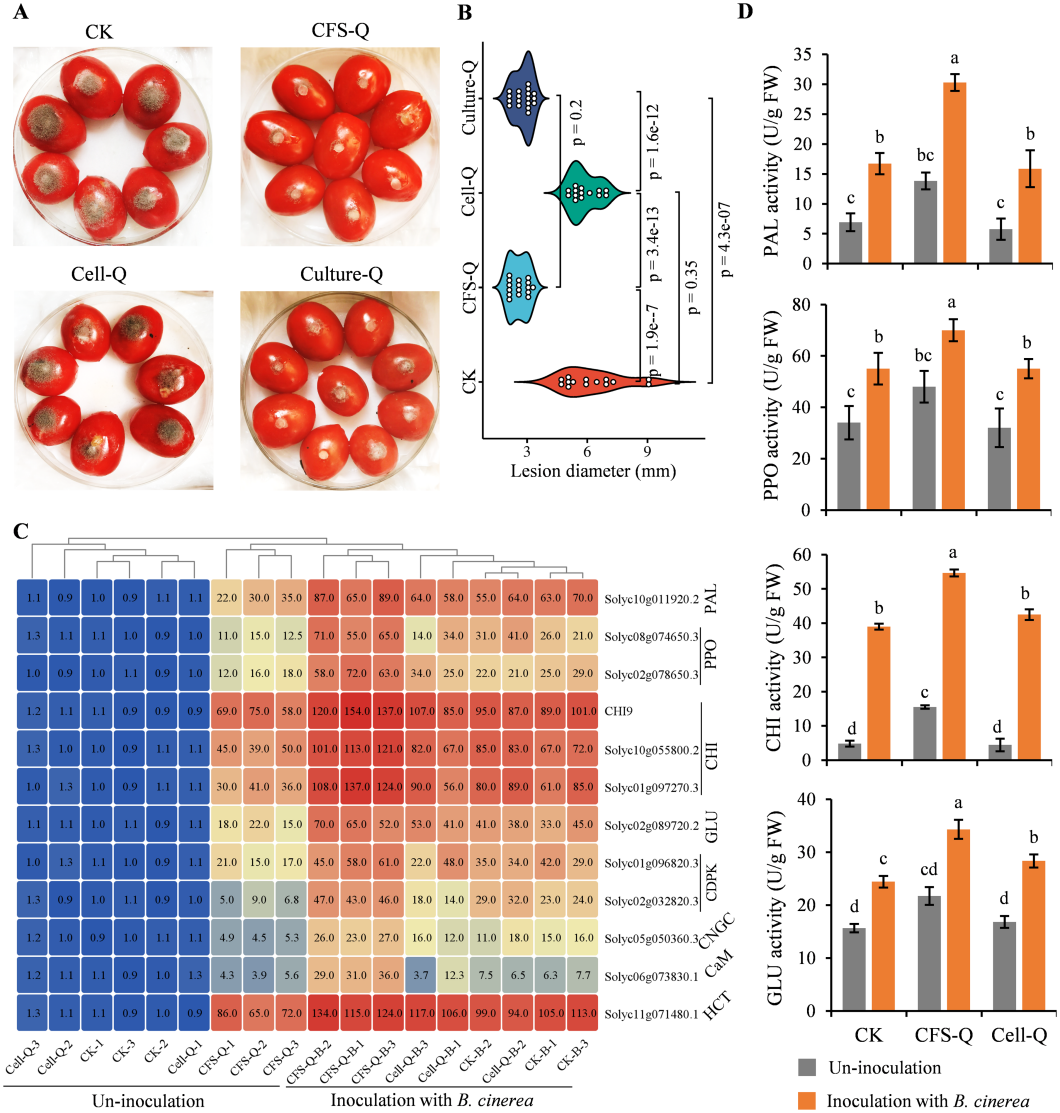
CFS-Q showed superior control efficacy in helping tomato fruits resist *B. cinerea* infection. **(A)** The disease phenotype on postharvest tomato fruits with different treatment. **(B)** The lesion size on postharvest tomato fruits with different treatment. **(C)** Heatmap showing the expression level of the indicated genes detected by RT-qPCR. SlACT was used as endogenous reference. The expression level of each gene in CK group was set as “1” and used as sample control. The value represents the fold change compared to CK. **(D)** activity of the indicated defense-related enzymes at 24 h post *B. cinerea* inoculation. The enzyme activity units are expressed on the fresh weight basis as U g^−1^. Different lowercase letters indicate a statistically significant difference (*p* < 0.01).

Tomato fruits were sprayed with CFS-Q, Cell-Q (OD_600_ = 0.1), and the fermentation broth of QSE-21 containing bacterial cells (Culture-Q) respectively. After 8 h, the *B. cinerea* was inoculated onto the fruits, and the disease lesion was measured after 96 h. Compared with the control group (treated with LB medium), the lesion areas caused by *B. cinerea* infection in the CFS-Q and Culture-Q treatment groups were significantly reduced, and there was no significant difference in the lesion area between these two treatments (Figures 7A,B). However, there was no significant difference in the lesion area caused by *B. cinerea* infection between the Cell-Q treatment group and the control group (Figures 7A,B). The expression levels of partial DEGs in the immune-related signaling pathways in tomato fruit flesh were detected at 24 h post-inoculation (hpi) with *B. cinerea*. Compared with the control group, the expression of the detected genes was significantly up-regulated in the CFS-Q and Culture-Q treatment groups (Figure 7C). In contrast, there was no significant difference in the expression levels of the most detected genes between the Cell-Q treatment group and the control group (Figure 7C). Meanwhile, the activities of the 4 defense-related enzymes (PAL, PPO, CHI, GLU) in the treated fruit flesh were measured. Compared with the control group, the activities of these 4 enzymes were significantly increased in the CFS-Q and Culture-Q treatment groups (Figure 7D). However, there was no significant difference in the activities of these 4 enzymes between the Cell-Q treatment group and the control group (Figure 7D). The above results suggest that the direct application CFS-Q can significantly enhance the induce the disease resistance of tomato fruits and improve their resistance to *B. cinerea* infection. The efficacy of CFS-Q in disease control is notable superior to that of using the cells of biocontrol bacteria (Cell-Q).

## 4 Discussion

The use of biological control agents (BCAs) has emerged as a promising alternative to chemical pesticides for managing plant diseases, offering environmentally friendly and sustainable solutions. However, the effectiveness of live microbial BCAs in field applications is often limited due to their non-native nature in ecological environments. *Bacillus velezensis*, a biocontrol bacterium that has recently garnered significant attention, has exhibited remarkable superiority in beneficial interactions with plants and the control of plant diseases (Kenfaoui et al., 2024; Su et al., 2024). It has been demonstrated that *B. velezensis* can secrete an array of substances into the cell-free supernatant (CFS) to directly inhibit the growth or invasion of pathogens, as well as to elicit systemic resistance in host plants (Kenfaoui et al., 2024). In this study, we explored the potential of CFS derived from *Bacillus velezensis* QSE-21 fermentation broth as an effective biocontrol agent against tomato gray mold disease.

### 4.1 The dual role of CFS-Q in assisting tomato to resist *Botrytis cinerea*

**Figure 8:**
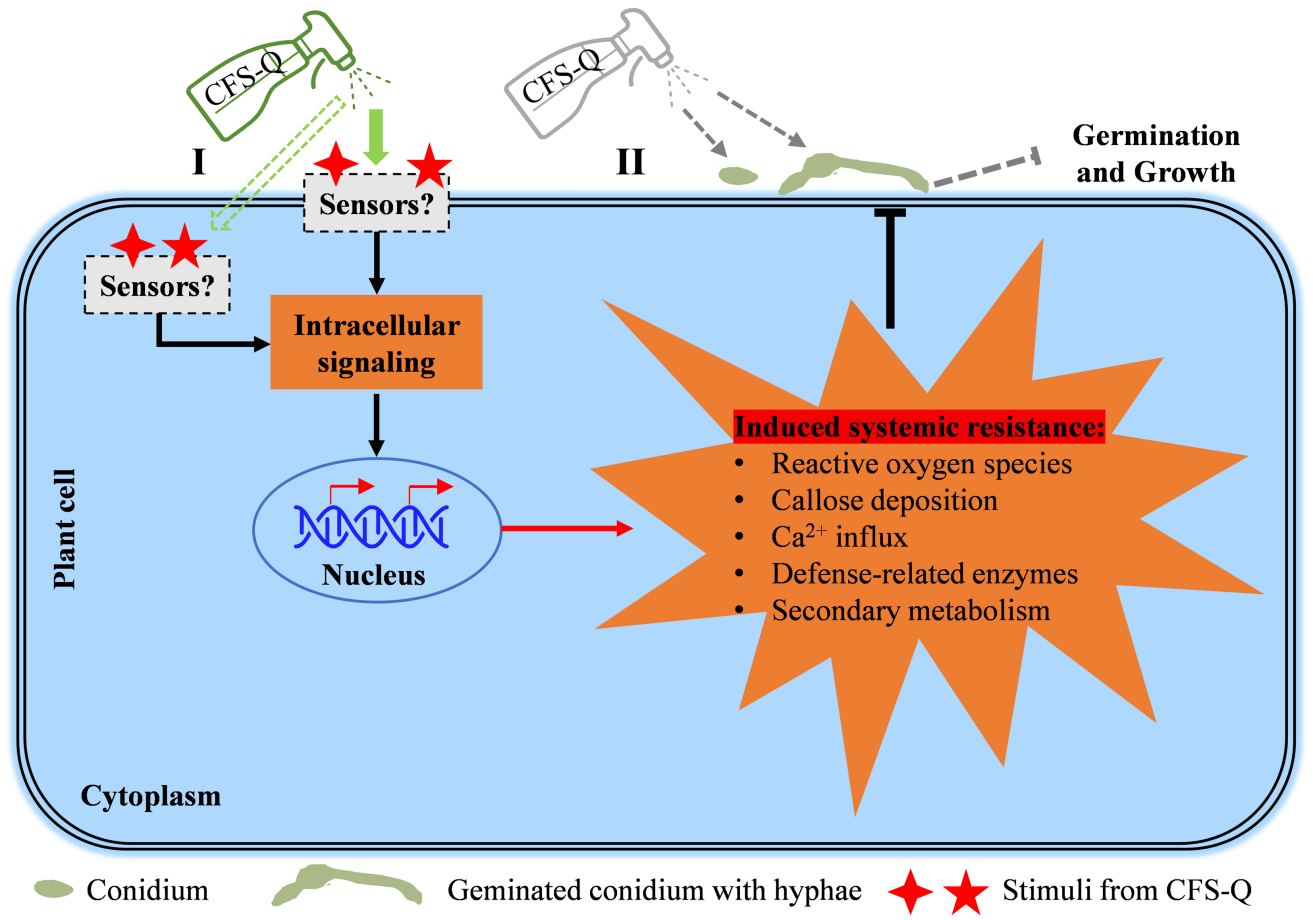
A working model of how CFS-Q helps plants resist *B. cinerea* infection. CFS-Q is capable of controlling *B. cinerea* both directly and indirectly. Sprayed CFS-Q stimulates plant intracellular immune signaling pathways through sensors on the plant cell membrane or in plant cells **(I)** and subsequently induces a range of early and late immune responses to *B. cinerea* infection. On the surface of plant **(II)**, sprayed CFS-Q comes in direct contact with the conidium and hyphae of *B. cinerea* to inhibit conidial germination and hyphal growth (previous study).

We have previously demonstrated that the CFS of QSE-21 (CFS-Q) can directly inhibit mycelial growth, conidial production and conidial germination of *B. cinerea* (Xu et al., 2021). In the present study, we further showed that CFS-Q can also induce immune responses in tomato plants and fruits. After treatment with CFS-Q, a series of immune responses, including rapid cell death, the accumulation of ROS, callose deposition, and upregulated expression of genes related to plant immunity, were activated in tomato leaves (Figure 1). Even without contact with *B. cinerea*, CFS-Q could still effectively help tomato resist infection by the pathogen (Figure 2A). In addition, pre-treating tomato leaves and fruits with CFS-Q enabled them to mount faster and stronger immune responses when perceiving the infection of *B. cinerea* (Figures 6, 7). Under the combined direct and indirect effects, CFS-Q can effectively prevent and control the occurrence of gray mold disease in tomato leaves and fruits (Figures 2, 7). Based on our previous and present research, we propose a working model for in which CFS-Q is involved in helping tomato resist *B. cinerea* infection (Figure 8). After being sprayed, CFS-Q can exert biocontrol control effects by inducing tomato defense responses and directly inhibiting the hyphal growth and conidial germination of *B. cinerea*. Therefore, CFS-Q has potential as a promising candidate for the development of biocontrol agents for the management of tomato gray mold disease.

This dual mode of action has been observed in various biocontrol microorganisms. For example, *Streptomyces* spp. can not only directly inhibit pathogenic fungi through the production of antibiotics, but also induce immune responses in plants (Le et al., 2021; Vergnes et al., 2020). *Bacillus* species, including *B. subtilis*, *B. amyloliquefaciens*, and *B. velezensis*, can directly inhibit pathogenic fungi by producing lipopeptides and cyclic peptides. Meanwhile, these peptide substances have also been proven to stimulate plant immune responses (Rahman et al., 2024; Xu et al., 2021). *Trichoderma* species primarily function through mycoparasitism, promoting plant growth, and enhancing plant immunity. However, they can also directly inhibit fungal growth by releasing substances such as hydrolytic enzymes and antibiotics (Kumari et al., 2025). BCAs function through dual or even multiple modes of action, exhibiting a diverse range of mechanisms. As a result, they are less likely to induce resistance even after long-term use.

### 4.2 CFS-Q induced immune priming confers enhanced systemic resistance in tomato

Upon treatment with CFS-Q, tomato seedlings exhibited typical immune responses, such as accumulation of reactive oxygen species (ROS), callose deposition, and activation of defense-related genes (Figure 1). ROS are crucial signaling molecules involved in plant defense mechanisms, playing roles in both direct antimicrobial activities and indirect modulation of downstream immune responses (Mittler et al., 2022). Callose is a polysaccharide that accumulates at plasmodesmata and strengthens cell walls as part of the plant’s defense strategy against pathogens (Wu et al., 2018). Thus, the induction of callose deposition by CFS-Q highlights its ability to fortify physical barriers within the plant.

Transcriptomic analysis revealed that CFS-Q treatment activated plant immunity signaling pathways, including defense-related secondary metabolism, hormone signaling, MAPK signaling, Ca^2+^ signaling, and defense-related enzymes (Figures 3–6; Supplementary Figure S4). Notably, with the treatment of CFS-Q, the expression of some key marker genes involved in plant immunity and Ca^2+^ signaling could respond more strongly to the infection of *B. cinerea* (Figure 6B). CFS-Q treatment induced the upregulated expression of the PAL genes and the increase of PAL enzyme activity (Figures 4, 5), which could guide the metabolic flux to phenylpropanoid metabolism (Dong and Lin, 2021; Zhang and Liu, 2014). CFS-Q treatment also induced the upregulated expression of the HCT genes and other genes related to lignin biosynthesis (Figure 4B; Supplementary Figure S2), suggesting that treatment with CFS-Q directed the metabolic flux toward lignin biosynthesis. Lignin accumulation can increase the mechanical strength of plant cell wall, thereby serving as an enhanced first line of defense against pathogen invasion (Kashyap et al., 2021; Wolf, 2022).

Calcium (Ca^2+^) is essential for plant processes and acts as a second messenger. Environmental stimuli increase cytoplasmic Ca^2+^ levels ([Ca^2+^]cyt), initiating signaling crucial for defense and symbiosis (Wang and Luan, 2024; Wang et al., 2022). CNGC-triggered Ca^2+^ influx is vital for both pattern-triggered immunity (PTI) and effector-triggered immunity (ETI) (Tian et al., 2019; Wang and Luan, 2024; Yuan et al., 2017). Protein sensors like CDPKs and CaMs decode Ca^2+^ signals, activating the downstream immune regulators such as ROS production by the respiratory burst oxidase homolog (Rboh) (Yuan et al., 2017). However, ROS accumulation in the absence of pathogens is extremely harmful to plants. Balancing downstream gene transcription is critical for maintaining the balance of plant growth and immunity. Our results showed that CFS-Q slightly increased [Ca^2+^]cyt and downstream gene expression (Figures 4C, 6), but the infection of pathogen induced higher transcription, especially in CFS-Q-treated leaves (Figure 6B). Furthermore, Ca^2+^ signal can be conducted over long distances in plant tissues (Choi et al., 2017). This might be the reason why we can detect enhanced immune responses and diseases resistance in distal tissues (Figures 2, 4). These results suggest that CFS-Q treatment can place plants in an immune priming state, thereby helping them resist pathogen infection.

### 4.3 Advantages of using CFS-Q over live BCAs

According to existing research, biocontrol microorganisms primarily utilize active substances secreted extracellularly to directly antagonize plant pathogens or indirectly stimulate immune responses in plants, thereby exerting the function of preventing plant diseases (Bonaterra et al., 2022; Boro et al., 2022; Chaudhary et al., 2024; Syed Ab Rahman et al., 2018). A large number of studies have shown that the use of fermentation broth of biocontrol bacteria alone can achieve good control effects on plant diseases (Le et al., 2021; Vergnes et al., 2020; Xiao et al., 2021; Zhang et al., 2022). Notably, our results showed that the direct application of CFS-Q exhibits superior control efficacy against *B. cinerea* in tomato fruits compared to the use of bacterial cells (Figure 7). The use of live BCAs requires the biocontrol bacteria to first achieve effective colonization before they can release active substances and exert their biological control functions, which leads to a lag in the application of live BCAs (Akum et al., 2021; Figueredo et al., 2017). In contrast, CFS containing large amounts of active substances can be obtained under stable indoor fermentation conditions, which can take effect rapidly in the direct application of CFS. This approach might be particularly advantageous in environments where bacterial colonization is unfavorable, such as on plant leaf surfaces or fruit surfaces. Additionally, during the early stages of disease development, the direct application of CFS can take effect more rapidly, thereby effectively controlling the spread of the disease. Moreover, the direct application of CFS, without introducing non-native living organisms into ecological environments, minimizes the potential risks associated with ecological invasions or negative environmental impacts caused live BCAs. Thus, bypassing the need for colonization ensures immediate efficacy and enhances the potential for successful biocontrol, the direct application of CFS is more advantageous than the use of live BCAs under specific conditions.

## 5 Conclusion

In conclusion, our study provided compelling evidence that the cell-free supernatant (CFS) derived from *B. velezensis* QSE-21 (CFS-Q) is a potent inducer of plant immunity against *B. cinerea*. Through functional and molecular mechanism analysis, we demonstrated that CFS-Q triggers immune priming state and thereby offers plant stronger and more rapid response to *B. cinerea* infection. In the control of tomato fruits gray mold, CFS-Q is superior to that of the live microbial cells. In combination with its faster and stronger direct antagonistic effects on *B. cinerea*, application of CFS-Q is a promising method for managing gray mold disease in plant and postharvest fruit. In the future research, we will focus on optimizing the production of CFS-Q and exploring its implications in different crops and various usage scenarios, further validating its potential as a viable alternative to chemical pesticides in agricultural practices.

## Data Availability

The datasets presented in this study can be found in online repositories. The names of the repository/repositories and accession number(s) can be found below: https://www.ncbi.nlm.nih.gov/, PRJNA1215988.

## Footnotes

^1^ http://www.ncbi.nlm.nih.gov/sra/

^2^ https://www.bioinformatics.com.cn/SRplot
