# Supplementary material for: Bacillus velezensis QSE-21 cell-free supernatant primes resistance and outperforms live cells in controlling Botrytis cinerea on tomato
Source: Front Microbiol. 2025 Aug 7;16:1639396. doi: 10.3389/fmicb.2025.1639396 (PMC12367801; doi:10.3389/fmicb.2025.1639396)
Supplement: Supplementary file 1 [file Table_1.DOCX]

***Bacillus velezensis* QSE-21 cell-free supernatant primes resistance and outperforms live cells in controlling** ***Botrytis cinerea* on tomato**

**Supplementary Materials**


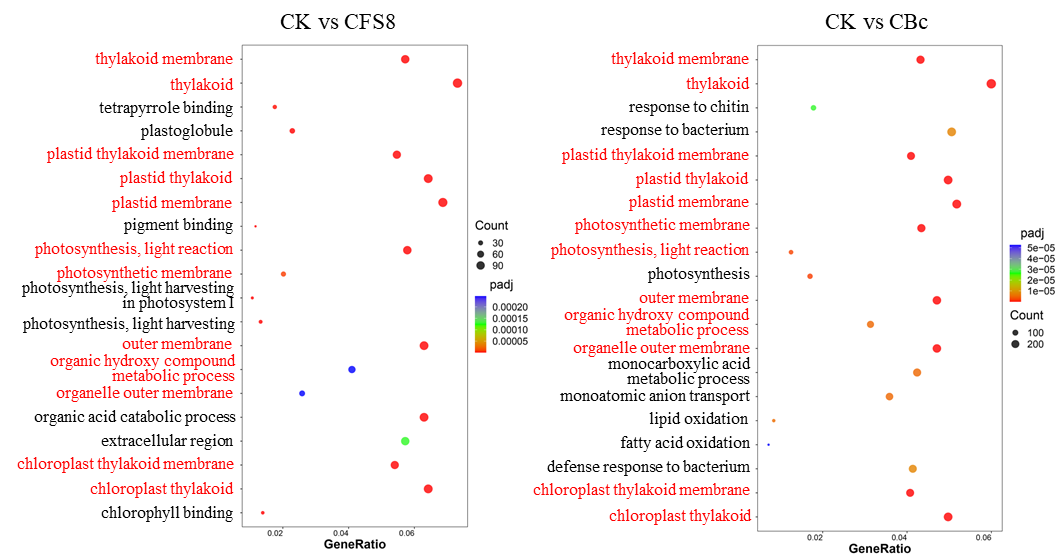


**Supplementary Figure 1.** Enrichment factor scatter plot of signifcant GO terms in tomato leaveas. The horizontal axis represents rich factors, and the vertical axis represents GO terms. The location of bubbles represents the enrichment item, the size of bubbles represents the number of DEGs, and the color of bubbles represents the significant degree of enrichment.


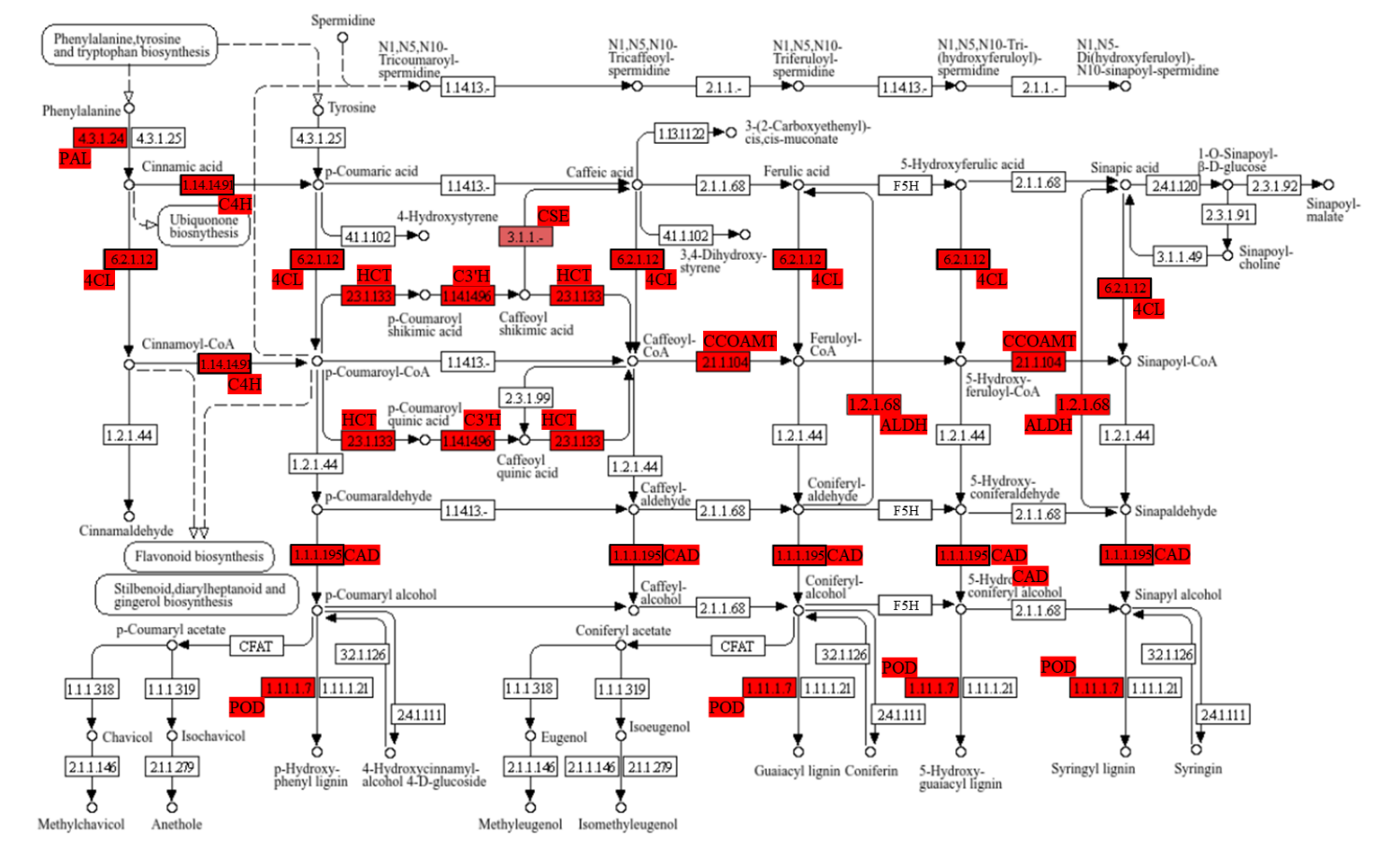


**Supplementary Figure 2.** Schematic diagram of Phenylpropanoid biosynthesis-*Solanum lycopersicum* (tomato) (PATHWAY: sly00940). The image was modified from KEGG Database (<https://www.kegg.jp/kegg/>). The steps marked in red indicate the steps where the up-regulated genes are located.


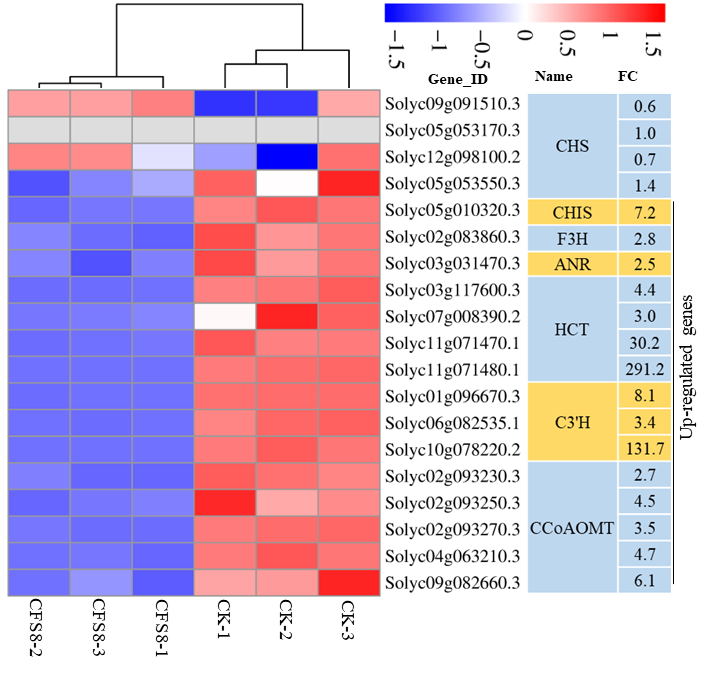


**Supplementary Figure 3.** Cluster heat maps of partial genes annotated to flavonoid biosynthesis pathway. CHS, Chalcone synthase. CHIS, Chalcone isomerase. F3H, Flavanone 3‐hydroxylase.


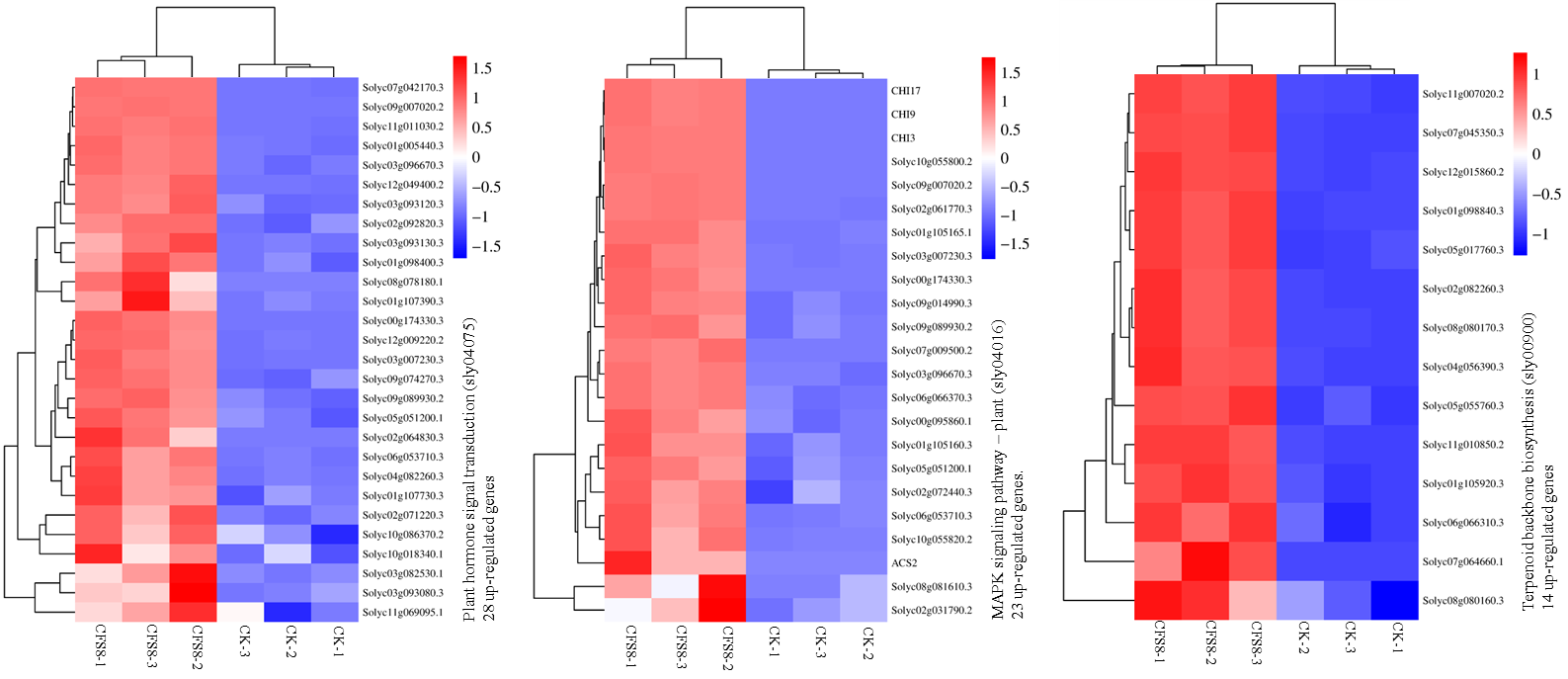


**Supplementary Figure 4.** Cluster heat maps of up-regulated genes annotated to plant hormone signal transduction pathway, MAPK signaling pathway-plant and terpenoid backbone biosynthesis pathway.


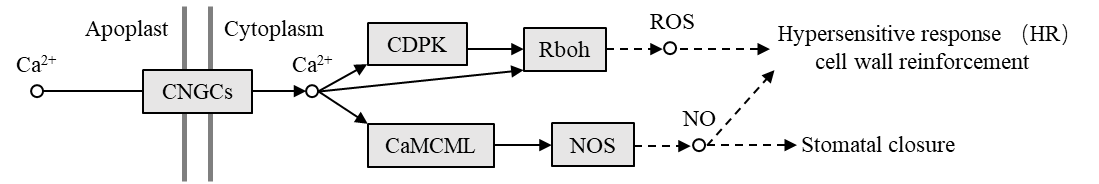


**Supplementary Figure 5.** Ca^2+^ signaling in the Plant-pathogen interaction pathway (sly04626). The image was modified from KEGG Database (<https://www.kegg.jp/kegg/>).

| **Supplementary Table 1.** Primers used for RT-qPCR in this paper. | | |
| --- | --- | --- |
| **Name** | **Forward primer (5` → 3`)** | **Reverse primer (5` → 3`)** |
| SlACT (Solyc04g011500) | TGGAATGGTCAAGGCTGG | TCAGTTAGGAGGACAGGATG |
| SlPTI5 (Solyc02g077370) | CCCAACGAAATCAATTACTC | CCCAACGAAATCAATTACTC |
| SlWRKY33 (Solyc09g014990) | CGAACCCAAGGAGTTACTA | TGTTGTTGTTGCTTCCAG |
| SlTD2 (Solyc09g008670) | CTCACATAATGAATTGGTTG | TTATATCTCCCTGATTACGGTA |
| SlPR1 (Solyc09g007020) | GATCCTGTAGCCATATTTCAC | TGGTAGCGTAGTTATAGTCTG |
| SlPR2 (Solyc01g059965) | AACACATTTCTTCTGGGATG | TTAATGTCATTTCCCAAACC |
| SlPR5 (Solyc08g080670) | ATGGGGTAAACCACCAAACA | AAGTGAACCAGGGCATTCAC |
| CHI9 (Solyc10g055810) | CTATCTGGTTCTGGATGAC | CGCAATACCTCCTGTAAA |
| Solyc05g050360 | GACGAACAATCACTGGAT | AGACTACTGGAGGAAGAAT |
| Solyc05g050350 | GACTTCTGTGGTGATGAG | ATGTCCTCCATTGTTGTG |
| Solyc01g096820 | GTGTTCTCAGCAGTAGTT | GTTCCATCACGATTGTTATC |
| Solyc02g032820 | TCACATCAGCAGAAGTTC | CCACTATTGTCCGTATCC |
| Solyc06g073830 | GGTAGGCGGAGATTTAAC | CTACTCAACATCCTCTTCAA |
| Solyc03g005040 | GAAGGATACAAGCGACTT | CCTCCATCTTCTACTAATCTC |
| Solyc10g011920 | CTTACTGCTCGGAACTTC | TTGCTACTTGGCTAACTG |
| Solyc08g074650 | CCTATACCGATACCTAATCTG | GCTTGAATGAACGAGGAT |
| Solyc02g078650 | GGTGCTTCTACTCTTGTT | CACTATCCACGGAATGAG |
| Solyc10g055800 | CTTAGAGAACAAGGTAGCC | GTCATCCAGAACCAGATAG |
| Solyc01g097270 | CGCTTACTGTTCAACTTG | TTCCCACTCCATCTGTAT |
| Solyc02g089720 | CAATTCACGCCATTCTTG | GCATTACCACTTGTAGGATA |
| Solyc01g096820 | GTGTTCTCAGCAGTAGTT | GTTCCATCACGATTGTTATC |
| Solyc02g032820 | TCACATCAGCAGAAGTTC | CCACTATTGTCCGTATCC |
| Solyc05g050360 | GACGAACAATCACTGGAT | AGACTACTGGAGGAAGAAT |
| Solyc06g073830 | GGTAGGCGGAGATTTAAC | CTACTCAACATCCTCTTCAA |
| Solyc11g071480 | CTCCTTCAACTACAACTCA | CGAACACCCTCATCATTA |
